# Precise staging of severe fever with thrombocytopenia syndrome: Integrating the extent of organ damage for prognostic and therapeutic applications

**DOI:** 10.1371/journal.pntd.0013249

**Published:** 2025-10-15

**Authors:** Yao Hao, Jing Sun, Qiongle Wu, Yingchun Sun, Zhiyu Pan, Xiaoyi Wang, Aiping Zhang, Manman Liang, Jianghua Yang

**Affiliations:** 1 Department of Infectious Diseases, Yijishan Hospital of Wannan Medical College, Wuhu, Anhui, People’s Republic of China; 2 Department of Cardiology, The First Affiliated Hospital of Nanjing Medical University, Nanjing, People’s Republic of China; Universiti Malaya, MALAYSIA

## Abstract

**Objectives:**

This study aimed to develop an accurate staging system for SFTS (Severe Fever with Thrombocytopenia Syndrome) based on the dynamic assessment of organ damage. This staging is intended to improve prognostication and guide treatment strategies.

**Methods:**

Clinical data and laboratory parameters were analyzed from 77 fatal and 398 non-fatal cases of SFTS. Risk factors for mortality were identified using univariate Cox regression analysis. Dynamic changes in laboratory parameters and multi-organ dysfunction were systematically observed.

**Results:**

By dynamically analyzing clinical symptoms and laboratory parameters over the disease course, combined with assessing the number and severity of multi-organ dysfunction, SFTS progression was categorized into five distinct stages: initial (1–4 days), progressive (5–7 days), MOD (Multiple Organ Dysfunction) (8–10 days), remission (11–14 days), and convalescence (15–20 days). The critical phase, lasting approximately two weeks, accounted for 85.71% of patients succumbed within this two-week period, with 46.75% experiencing mortality during the MOD stage. Moreover, the study findings highlighted the effectiveness of intravenous immunoglobulin, including both its overall and early administration, in improving outcomes for patients with SFTS-associated myocarditis.

**Conclusions:**

The progression of SFTS can be distinctly categorized into five distinct stages. Day 7 represents a critical juncture in disease progression, whereas Day 11 signifies a pivotal moment for clinical recovery. The progression stage is optimal for intervening to prevent further disease advancement. Treatment strategies should be adapted to the evolving patterns and severity of organ dysfunction. Myocarditis remains a significant challenge throughout SFTS progression, and early IVIG administration has been demonstrated to reduce mortality in patients with myocarditis complications significantly.

## Author summary

Multi-organ dysfunction (MOD) is a dynamic process, with myocardial injury persisting throughout the course of disease. Myocarditis has been identified as a significant risk factor for mortality in patients with SFTS. The progression of MOD in SFTS is dynamic, with myocardial injury persisting throughout the disease course. It was found in this study that the progression of SFTS can be systematically categorized into five distinct stages. Day 7 marks a critical juncture in disease progression, while Day 11 represents a pivotal moment for clinical recovery. The progression stage is the optimal period for intervention to prevent further disease advancement. Treatment strategies should be tailored to the evolving patterns and severity of organ dysfunction. Myocarditis poses a significant challenge throughout the course of SFTS, and early administration of intravenous immunoglobulinhas been shown to reduce mortality in patients with myocarditis complications substantially.

## Introduction

Severe fever with thrombocytopenia syndrome (SFTS) is an acute, rapidly progressing infectious disease caused by the Dabie Bandavirus. With a mortality rate ranging from 10% to 20%, its incidence has been increasing annually [1–7]. Early-stage SFTS lacks specific clinical manifestations, complicating clinical diagnosis. The World Health Organization (WHO) has classified SFTS as a high-priority infectious disease for further research due to its substantial fatality rate and pandemic potential, posing a significant public health risk [8]. Current research on SFTS staging primarily adopts either a three-stage or four-stage framework. Gai et al. [9] classified SFTS progression into three stages based on laboratory parameter dynamics: the fever stage (first week), the multi-organ dysfunction (MOD) stage (7–13 days after onset), and the convalescence stage (two weeks post-onset) [9]. Subsequent studies have refined the three-stage staging model [10,11]. Conversely, Weng et al. [12] proposed a four-stage classification: 1–7 days, 8–10 days, 11–13 days, and 14 days or beyond, identifying the acute phase as the first 14 days of illness. Regardless of the staging method employed, the early course of SFTS remains inadequately studied through dynamic observation, limiting the ability to capture disease progression accurately. Furthermore, laboratory parameters are neither classified by functional categories nor integrated with MOD. The absence of a dynamic framework for assessing changes in organ function limits the ability to develop and implement stage-specific treatment strategies that adapt to evolving patient needs.

SFTS progresses rapidly, as outlined in the 2023 national guidelines for SFTS diagnosis and treatment. Patients are classified into four categories: mild, moderate, severe, and critical [13]. Mild cases typically manifest symptoms such as fever, thrombocytopenia, fatigue, dizziness, headache, nausea, vomiting, and diarrhea. However, in patients with critical illness, the clinical condition can deteriorate rapidly, resulting in multiple organ failure (myocardial injury, renal and hepatic impairment, electrolyte imbalances, and disseminated intravascular coagulation [DIC]) [14–16], and ultimately death. Research indicates that myocarditis may be a significant risk factor for mortality in patients with SFTS [17]. Multi-organ dysfunction (MOD) is a dynamic process, with myocardial injury persisting throughout the course of disease. The prognosis of patients with SFTS complicated with myocarditis appears to be closely tied to this condition. Intravenous immunoglobulin (IVIG) has demonstrated efficacy in treating myocarditis, but its therapeutic role in SFTS complicated by myocarditis remains unclear and requires further investigation [18–21].

Myocarditis has been identified as a significant risk factor for mortality in patients with SFTS. The progression of MOD in SFTS is dynamic, with myocardial injury persisting throughout the disease course. While IVIG has shown efficacy in treating myocarditis, its effectiveness in patients with SFTS and concurrent myocarditis remains uncertain. This variability in efficacy may stem from differing disease phases and the timing of intervention. It is crucial to develop an accurate method for tracking disease progression, clarifying the dynamics of MOD, and tailoring early interventions based on specific patterns of organ function impairment. Therefore, a comprehensive study involved 475 patients with SFTS, documenting their clinical manifestations and laboratory findings across all disease stages. Furthermore, the disease progression and IVIG treatment outcomes were analyzed in 134 patients with SFTS complicated by myocarditis.

## Result

### General information

**Fig 1:**
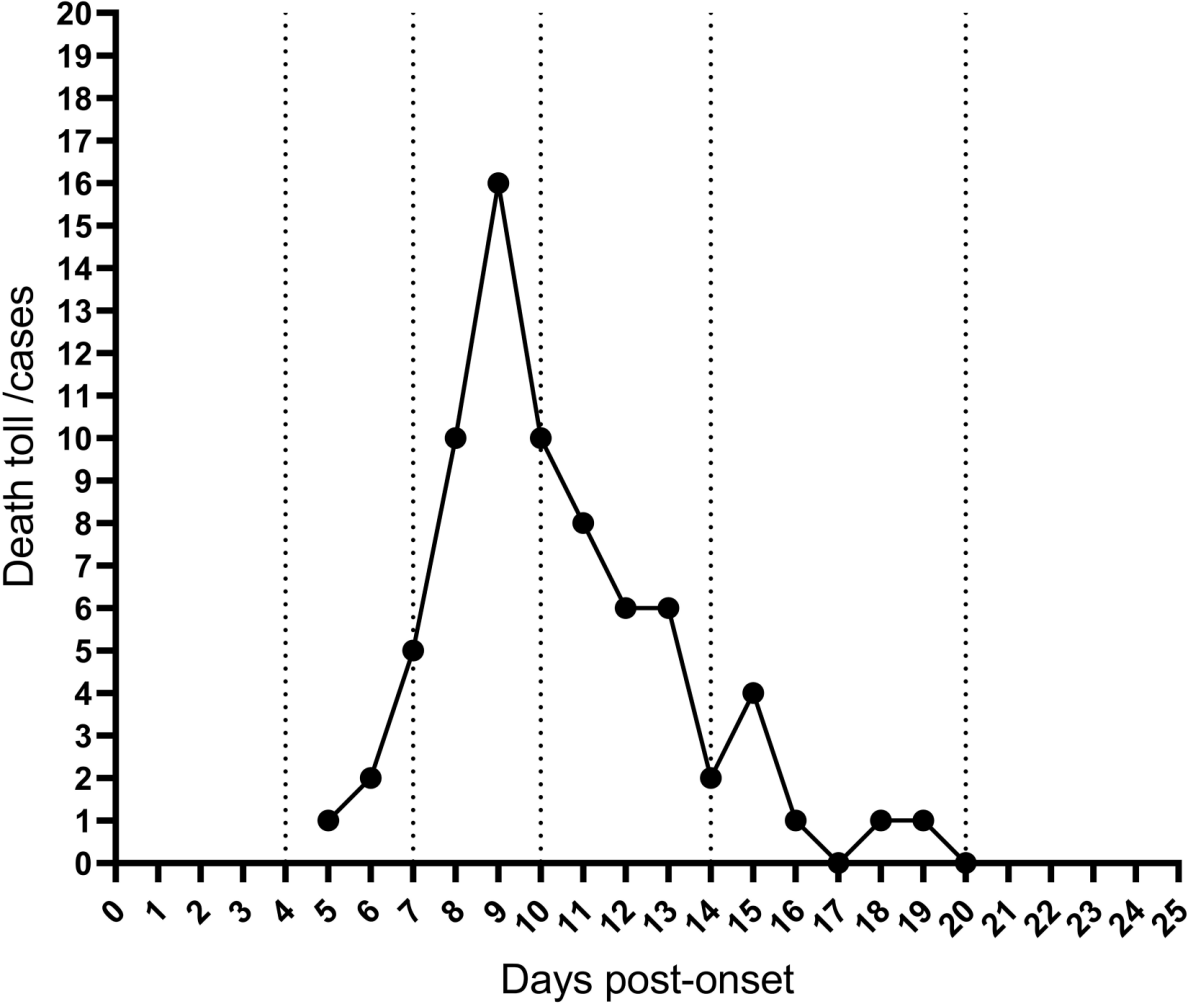
Time from onset to death in the fatal group.

The study cohort comprised 475 patients diagnosed with SFTS, comprising 208 men (43.79%) and 267 women (56.21%) (p = 0.567). Among these, 77 patients (16.21%) succumbed to the disease (fatal group), while 398 patients (83.79%) survived (non-fatal group). The mean age of the non-fatal group was 63.60 ± 10.46 years, compared to 69.53 ± 12.16 years in the fatal group (p = 0.206). General characteristics and clinical symptoms of patients with SFTS are summarized in Table 1. No significant differences were observed between the fatal and non-fatal groups regarding age, sex, or history of tick bites. However, significant differences were noted in the presence of fatigue (p < 0.001), headache (p = 0.040), and neurological symptoms (p = 0.002). Analysis of the time from symptom onset to death in the fatal group (Fig 1) revealed no mortalities within the first 4 days of the disease onset. Mortality began to rise between Days 5 and 7, with eight fatalities recorded. The highest mortality occurred between Days 8 and 10, during which 36 patients (46.75%) succumbed to the disease. Overall, 85.71% of fatalities occurred within the first two weeks of disease onset. (Fig 1).

**Table 1:** Clinical Features in Fatal and Non-fatal group.

	Total	Fatal group	Non-fatal group	P^a^
	(N = 475)	(N = 77)	(N = 398)	
**General information**				
Gender (Male/Female)	(208/267)	(36/41)	(172/226)	0.567
Age, years	65.12 ± 10.64	66.35 ± 10.93	64.88 ± 10.58	0.206
History of tick bites (%)	112 (23.58)	19 (24.67)	93 (23.37)	0.804
**Symptoms and signs**				
Fever (%)	434 (91.36)	70 (90.91)	364 (91.46)	0.875
Myalgia (%)	88 (18.53)	10 (12.99)	78 (19.60)	0.172
Fatigue (%)	344 (72.42)	38 (49.35)	306 (76.88)	<0.001
Cough and Sputum (%)	92 (19.37)	17 (22.08)	75 (18.84)	0.511
Abdominal pain (%)	59 (12.42)	13 (16.88)	46 (11.56)	0.195
Vomiting and Nausea (%)	116 (24.42)	17 (22.08)	99 (24.87)	0.601
Headache (%)	58 (12.21)	4 (5.19)	54 (13.57)	0.040
Neurological symptoms (%)	26 (5.47)	10 (12.99)	16 (4.02)	0.002

^a^Mann–Whitney U test was used to compare qualitative variables. p < 0.05 was considered statistically significant.

### Laboratory parameter analysis

#### Risk assessment for mortality.

**Fig 2:**
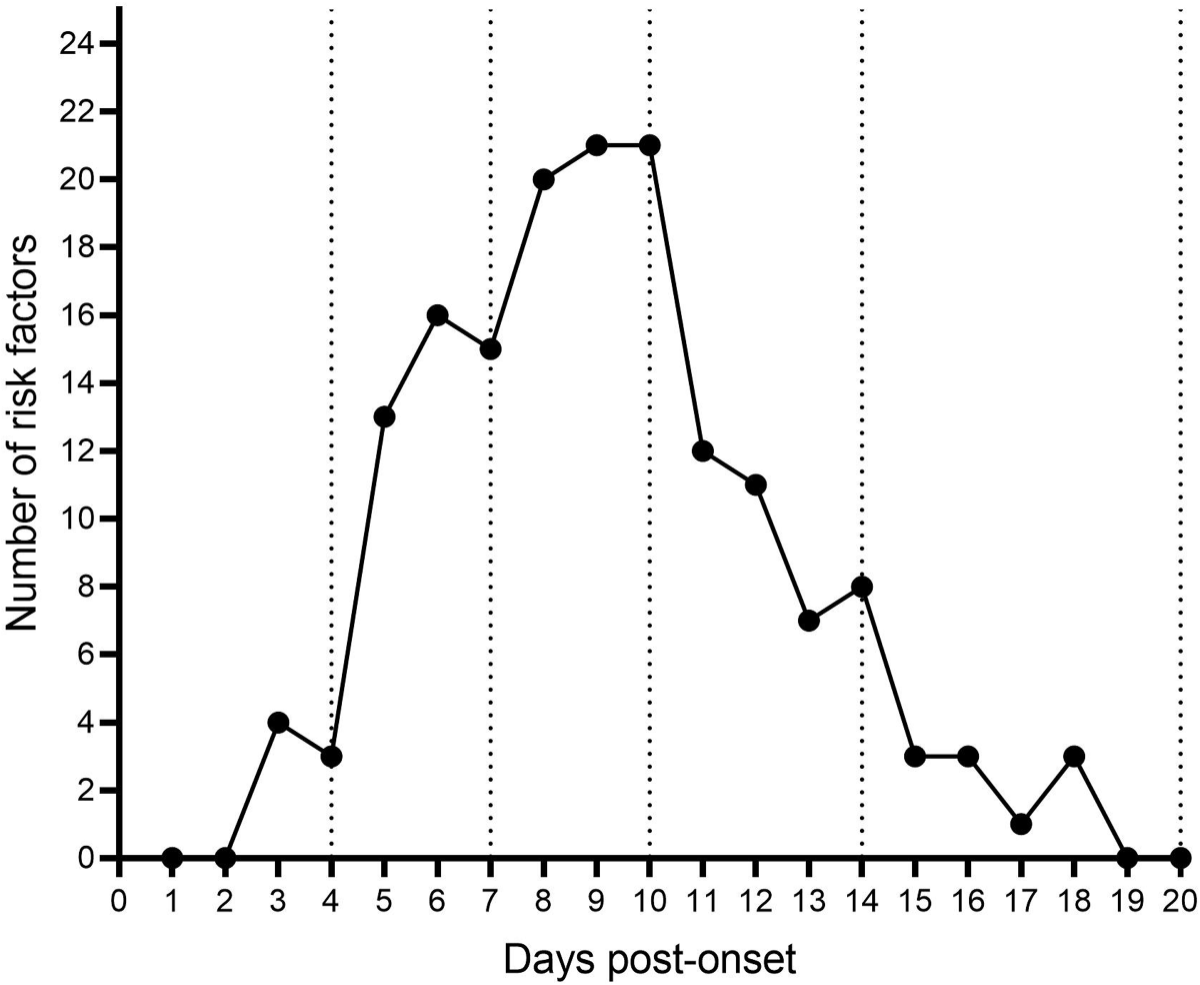
Number of risk factors in the course of the disease.

To evaluate the extent and progression of MOD in patients with SFTS, a dynamic univariate Cox regression analysis was performed on laboratory parameters collected during the first 20 days after the disease onset (Table 2). The analysis indicated that the number of risk factors associated with mortality increased from Day 3, peaking on Day 9, and began to decline from Day 11, eventually reaching zero by Day 19. A graph combining the number of risk factors with disease duration was plotted to facilitate a more intuitive understanding (Fig 2). Analyzing Fig 1 alongside Fig 2 revealed a consistent trend between daily mortality rates and the number of risk factors. Both increased gradually during the first 4 days, accelerated between Days 5–7, peaked sharply between Days 8–10, decreased from Days 11–14, and eventually dropped to zero between Days 15–20 of the disease course.

**Table 2:** Risk factors associated with the death of patients with SFTS.

Time	Parameters	β	SE	Wald2	p Value	OR (95% CI)
Day 3	ALT (U/L)	0.011	0.005	4.444	0.035	1.011 (1.001-1.021)
	AST (U/L)	0.003	0.001	4.852	0.028	1.003 (1.000-1.006)
	SCR (umol/L)	0.016	0.008	3.831	0.050	1.017 (1.000-1.033)
	cTn (ug/L)	30.465	14.551	4.384	0.036	1.701E + 13 (7.000-4.137E + 25)
Day 4	BUN (mmol/L)	0.000	0.000	4.173	0.041	1.000 (1.000-1.001)
	PCT (ng/ml)	0.200	0.085	5.612	0.018	1.222 (1.035-1.442)
	Myoglobin (ng/ml)	0.006	0.002	5.749	0.017	1.006 (1.001-1.010)
Day 5	PLT (10^9/L)	-0.020	0.008	6.264	0.012	0.980 (0.965-0.996)
	ALT (U/L)	0.007	0.002	9.996	0.002	1.007 (1.003-1.011)
	AST (U/L)	0.001	0.000	16.921	0.000	1.001 (1.001-1.002)
	BUN (mmol/L)	0.022	0.008	7.684	0.006	1.022 (1.006-1.038)
	CKMB (U/L)	0.022	0.007	9.421	0.002	1.022 (1.008-1.036)
	LDH (U/L)	0.001	0.000	11.318	0.001	1.001 (1.000-1.001)
	HBDH (U/L)	0.001	0.001	6.274	0.012	1.001 (1.000-1.002)
	amylase (U/L)	0.004	0.001	7.866	0.005	1.004 (1.001-1.007)
	APTT (S)	0.050	0.016	10.341	0.001	1.052 (1.020-1.085)
	TT (S)	0.021	0.005	15.450	0.000	1.022 (1.011-1.085)
	PLT (10^9/L)	0.307	0.112	7.545	0.006	1.360 (1.092-1.694)
	cTn (ug/L)	1.348	0.458	8.672	0.003	3.850 (1.570-9.442)
	Myoglobin (ng/ml)	0.001	0.000	10.956	0.001	1.001 (1.000-1.002)
Day 6	RBC (10^12/L)	0.708	0.281	6.344	0.012	2.030 (1.170-3.521)
	Hgb (g/L)	0.022	0.009	6.133	0.013	1.022 (1.005-1.040)
	PLT (10^9/L)	-0.031	0.008	13.040	0.000	0.970 (0.954-0.986)
	ALT (U/L)	0.002	0.000	11.936	0.001	1.002 (1.001-1.003)
	AST (U/L)	0.001	0.000	22.486	0.000	1.001 (1.000-1.001)
	BUN (mmol/L)	0.106	0.031	11.444	0.001	1.111 (1.045-1.182)
	SCR (umol/L)	0.011	0.002	19.247	0.000	1.011 (1.006-1.016)
	LDH (U/L)	0.000	0.000	23.445	0.000	1.000 (1.000-1.000)
	HBDH (U/L)	0.002	0.001	6.542	0.011	1.002 (1.000-1.003)
	K (+) (mmol/L)	0.758	0.323	5.519	0.019	2.135 (1.134-4.020)
	Ca (2+) (mmol/L)	-4.368	1.139	14.692	0.000	0.013 (0.001-0.118)
	PT (S)	0.096	0.026	13.779	0.000	1.101 (1.046-1.158)
	APTT (S)	0.047	0.007	43.006	0.000	1.048 (1.034-1.063)
	TT (S)	0.021	0.004	23.252	0.000	1.021 (1.021-1.029)
	D-Dimer (mg/L)	0.049	0.015	11.278	0.001	1.050 (1.021-1.081)
	BNP (pg/mL)	0.000	0.000	7.929	0.005	1.000 (1.000-1.000)
Day 7	PLT (10^9/L)	-0.017	0.008	4.460	0.035	0.983 (0.968-0.999)
	CRP (mg/L)	0.018	0.008	4.991	0.025	1.018 (1.002-1.035)
	AST (U/L)	0.002	0.000	34.948	0.000	1.002 (1.001-1.002)
	BUN (mmol/L)	0.093	0.025	14.348	0.000	1.098 (1.046-1.152)
	SCR (umol/L)	0.009	0.002	22.139	0.000	1.009 (1.005-1.013)
	Cystatin‐C (mg/L)	0.729	0.355	4.204	0.040	2.073 (1.033-4.159)
	CKMB (U/L)	0.007	0.003	5.457	0.019	1.007 (1.001-1.014)
	LDH (U/L)	0.001	0.000	27.158	0.000	1.001 (1.000-1.001)
	Cl (-) (mmol/L)	0.061	0.029	4.244	0.039	1.062 (1.003-1.125)
	APTT (S)	0.041	0.007	32.218	0.000	1.041 (1.027-1.056)
	TT (S)	0.015	0.003	18.499	0.000	1.015 (1.008-1.022)
	D-Dimer (mg/L)	0.045	0.010	22.097	0.000	1.046 (1.027-1.066)
	BNP (pg/mL)	0.000	0.000	10.328	0.001	1.000 (1.000-1.000)
	Myoglobin (ng/ml)	0.001	0.000	10.913	0.001	1.001 (1.000-1.001)
	Ferritin (ug/L)	0.000	0.000	4.764	0.029	1.000 (1.000-1.000)
Day 8	PLT (10^9/L)	-0.020	0.007	7.401	0.007	0.980 (0.996-0.994)
	CRP (mg/L)	0.031	0.006	31.014	0.000	1.031 (1.020-1.042)
	ALT (U/L)	0.002	0.001	4.098	0.043	1.002 (1.000-1.003)
	AST (U/L)	0.001	0.000	20.270	0.000	1.001 (1.001-1.001)
	BUN (mmol/L)	0.122	0.020	35.671	0.000	1.130 (1.085-1.176)
	SCR (umol/L)	0.011	0.001	52.700	0.000	1.011 (1.008-1.014)
	Cystatin‐C (mg/L)	0.347	0.160	4.728	0.030	1.415 (1.035-1.934)
	CK (U/L)	0.000	0.000	15.947	0.000	1.000 (1.000-1.000)
	CKMB (U/L)	0.001	0.000	13.058	0.000	1.001 (1.001-1.002)
	LDH (U/L)	0.001	0.000	36.955	0.000	1.001 (1.000-1.001)
	HBDH (U/L)	0.002	0.000	22.129	0.000	1.002 (1.001-1.003)
	PT (S)	0.051	0.024	4.629	0.031	1.052 (1.005-1.102)
	APTT (S)	0.043	0.007	33.779	0.000	1.044 (1.029-1.060)
	TT (S)	0.011	0.003	10.132	0.001	1.011 (1.004-1.017)
	D-Dimer (mg/L)	0.032	0.012	7.288	0.007	1.032 (1.009-1.056)
	BNP (pg/mL)	0.000	0.000	10.660	0.001	1.000 (1.000-1.000)
	PCT (ng/ml)	0.035	0.010	12.497	0.000	1.036 (1.016-1.057)
	Myoglobin (ng/ml)	0.001	0.000	32.021	0.000	1.001 (1.001-1.002)
	Ferritin (ug/L)	0.000	0.000	8.757	0.003	1.000 (1.000-1.000)
	IL-6 (pg/mL)	0.004	0.001	16.084	0.000	1.004 (1.002-1.005)
Day 9	RBC (10^12/L)	-0.387	0.193	4.035	0.045	0.679 (0.465-0.991)
	HCT	-0.022	0.009	6.808	0.009	0.978 (0.962-0.994)
	CRP (mg/L)	0.026	0.007	13.838	0.000	1.027 (1.012-1.041)
	AST (U/L)	0.001	0.000	26.908	0.000	1.001 (1.001-1.002)
	BUN (mmol/L)	0.027	0.012	5.091	0.024	1.027 (1.004-1.051)
	SCR (umol/L)	0.009	0.002	25.208	0.000	1.009 (1.005-1.012)
	Cystatin‐C (mg/L)	0.935	0.335	7.784	0.005	2.546 (1.321-4.910)
	CK (U/L)	0.000	0.000	7.401	0.007	1.000 (1.000-1.000)
	CKMB (U/L)	0.020	0.003	39.694	0.000	1.021 (1.014-1.027)
	LDH (U/L)	0.000	0.000	32.346	0.000	1.000 (1.000-1.000)
	HBDH (U/L)	0.001	0.000	18.069	0.000	1.001 (1.001-1.002)
	K (+) (mmol/L)	0.642	0.171	14.154	0.000	1.901 (1.360-2.656)
	PT (S)	0.165	0.028	33.546	0.000	1.179 (1.115-1.247)
	APTT (S)	0.049	0.007	45.742	0.000	1.051 (1.036-1.066)
	TT (S)	0.012	0.003	12.496	0.000	1.012 (1.005-1.019)
	D-Dimer (mg/L)	0.061	0.020	9.759	0.002	1.063 (1.023-1.105)
	BNP (pg/mL)	0.000	0.000	5.686	0.017	1.000 (1.000-1.000)
	PCT (ng/ml)	0.043	0.016	7.424	0.006	1.044 (1.012-1.077)
	cTn (ug/L)	0.375	0.093	16.331	0.000	1.455 (1.213-1.745)
	Myoglobin (ng/ml)	0.001	0.000	12.033	0.001	1.001 (1.000-1.001)
	IL-6 (pg/mL)	0.001	0.001	5.562	0.018	1.001 (1.000-1.002)
Day 10	WBC (10^9/L)	0.088	0.034	6.521	0.011	1.092 (1.021-1.168)
	Neutrophils (10^9/L)	0.116	0.043	7.147	0.008	1.123 (1.031-1.223)
	MPV (fl)	-0.373	0.155	5.773	0.016	0.689 (0.508-0.934)
	AST (U/L)	0.001	0.000	9.730	0.002	1.001 (1.000-1.002)
	BUN (mmol/L)	0.094	0.019	23.376	0.000	1.098 (1.057-1.141)
	SCR (umol/L)	0.009	0.002	25.720	0.000	1.009 (1.006-1.013)
	Cystatin‐C (mg/L)	0.764	0.340	5.053	0.025	2.147 (1.103-4.179)
	CK (U/L)	0.000	0.000	18.248	0.000	1.000 (1.000-1.000)
	CKMB (U/L)	0.013	0.002	28.185	0.000	1.013 (1.008-1.018)
	LDH (U/L)	0.001	0.000	13.815	0.000	1.001 (1.000-1.001)
	HBDH (U/L)	0.001	0.000	9.200	0.002	1.001 (1.000-1.001)
	K (+) (mmol/L)	1.009	0.213	22.413	0.000	2.743 (1.806-4.165)
	Cl (-) (mmol/L)	-0.021	0.008	6.287	0.012	0.980 (0.964-0.996)
	PT (S)	0.226	0.044	27.037	0.000	1.254 (1.152-1.366)
	APTT (S)	0.026	0.007	12.592	0.000	1.026 (1.012-1.041)
	TT (S)	0.009	0.004	5.164	0.023	1.009 (1.001-1.017)
	D-Dimer (mg/L)	0.070	0.018	15.519	0.000	1.072 (1.036-1.110)
	BNP (pg/mL)	0.000	0.000	3.980	0.046	1.000 (1.000-1.000)
	PCT (ng/ml)	0.233	0.058	16.055	0.000	1.262 (1.126-1.414)
	cTn (ug/L)	0.484	0.107	20.303	0.000	1.622 (1.314-2.003)
	Myoglobin (ng/ml)	0.001	0.000	17.933	0.000	1.001 (1.001-1.001)
Day 11	MCHC (g/L)	0.043	0.013	10.243	0.001	1.044 (1.017-1.071)
	RDW (%)	0.312	0.093	11.230	0.001	1.367 (1.138-1.640)
	AST (U/L)	0.001	0.000	4.815	0.028	1.001 (1.000-1.001)
	BUN (mmol/L)	0.077	0.029	7.125	0.008	1.080 (1.021-1.143)
	SCR (umol/L)	0.005	0.002	11.051	0.001	1.005 (1.002-1.008)
	CKMB (U/L)	0.009	0.003	11.531	0.001	1.010 (1.004-1.015)
	LDH (U/L)	0.001	0.000	22.051	0.000	1.001 (1.000-1.001)
	HBDH (U/L)	0.001	0.000	16.122	0.000	1.001 (1.001-1.002)
	Lipase (U/L)	0.001	0.000	5.864	0.015	1.001 (1.000-1.002)
	APTT (S)	0.042	0.008	28.609	0.000	1.043 (1.027-1.059)
	Myoglobin (ng/ml)	0.001	0.000	7.848	0.005	1.001 (1.000-1.001)
	Ferritin (ug/L)	0.000	0.000	8.082	0.004	1.000 (1.000-1.000)
Day 12	MCHC (g/L)	0.042	0.019	5.030	0.025	1.043 (1.005-1.083)
	MPV (fl)	-0.462	0.232	3.948	0.047	0.630 (0.399-0.994)
	AST (U/L)	0.001	0.000	6.146	0.013	1.001 (1.000-1.002)
	CKMB (U/L)	0.015	0.005	8.744	0.003	1.015 (1.005-1.025)
	LDH (U/L)	0.000	0.000	25.483	0.000	1.000 (1.000-1.000)
	HBDH (U/L)	0.001	0.000	10.395	0.001	1.001 (1.001-1.002)
	Cl (-) (mmol/L)	-0.026	0.013	4.084	0.043	0.974 (0.950-0.999)
	Ca (2+) (mmol/L)	0.030	0.010	8.637	0.003	1.030 (1.010-1.050)
	PT (S)	0.179	0.047	14.381	0.000	1.196 (1.090-1.311)
	APTT (S)	0.062	0.013	20.950	0.000	1.064 (1.036-1.092)
	cTn (ug/L)	0.851	0.236	13.000	0.000	2.341 (1.474-3.718)
Day 13	MCHC (g/L)	0.045	0.017	7.503	0.006	1.046 (1.013-1.081)
	PLT (10^9/L)	-0.035	0.014	6.708	0.010	0.965 (0.940-0.991)
	LDH (U/L)	0.002	0.000	10.507	0.001	1.002 (1.001-1.002)
	CO2CP (mmol/L)	-0.230	0.105	4.767	0.029	0.795 (0.647-0.977)
	amylase (U/L)	0.001	0.001	6.290	0.012	1.001 (1.000-1.002)
	D-Dimer (mg/L)	0.064	0.026	6.104	0.013	1.066 (1.013-1.121)
	BNP (pg/mL)	0.000	0.000	10.836	0.001	1.000 (1.000-1.000)
Day 14	ALT (U/L)	0.013	0.006	3.850	0.050	1.013 (1.000-1.026)
	AST (U/L)	0.004	0.001	8.821	0.003	1.004 (1.001-1.006)
	SCR (umol/L)	0.012	0.003	12.374	0.000	1.012 (1.005-1.019)
	CK (U/L)	0.002	0.001	11.302	0.001	1.002 (1.001-1.003)
	CKMB (U/L)	0.074	0.024	9.360	0.002	1.077 (1.027-1.129)
	Ca (2+) (mmol/L)	-4.408	2.124	4.307	0.038	0.012 (0.000-0.783)
	CO2CP (mmol/L)	-0.238	0.105	5.204	0.023	0.788 (0.642-0.967)
	PCT (ng/ml)	2.995	1.120	7.149	0.007	19.977 (2.224-179.414)
Day15	Hemoglobin (g/L)	0.040	0.020	4.233	0.040	1.041 (1.002-1.082)
	SCR (umol/L)	0.008	0.004	4.852	0.028	1.008 (1.001-1.015)
	D-Dimer (mg/L)	0.083	0.042	3.888	0.049	1.087 (1.000-1.180)
Day 16	RBC (10^12/L)	1.507	0.742	4.126	0.042	4.514 (1.054-19.325)
	Hemoglobin (g/L)	0.051	0.024	4.478	0.034	1.052 (1.004-1.103)
	SCR (umol/L)	0.010	0.005	3.936	0.047	1.010 (1.000-1.021)
Day 17	MCHC (g/L)	0.163	0.081	4.033	0.045	1.177 (1.004-1.380)
Day 18	CKMB (U/L)	0.225	0.105	4.605	0.032	1.253 (1.020-1.539)
	Cl (-) (mmol/L)	-0.655	0.324	4.084	0.043	0.519 (0.275-0.980)

### Dynamic profile of laboratory parameters

**Fig 3:**
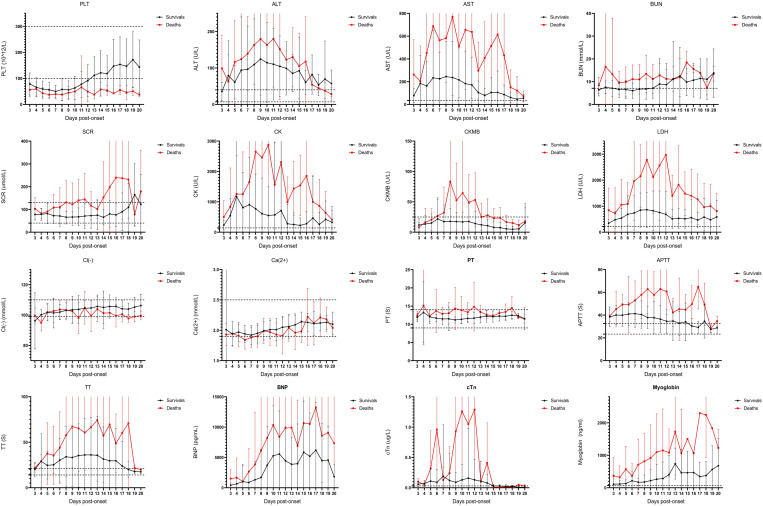
Dynamic profile of laboratory parameters. (A) A line chart was made to compare the changes of the two groups of patients with the main risk factors in univariate analysis.

**Fig 4:**
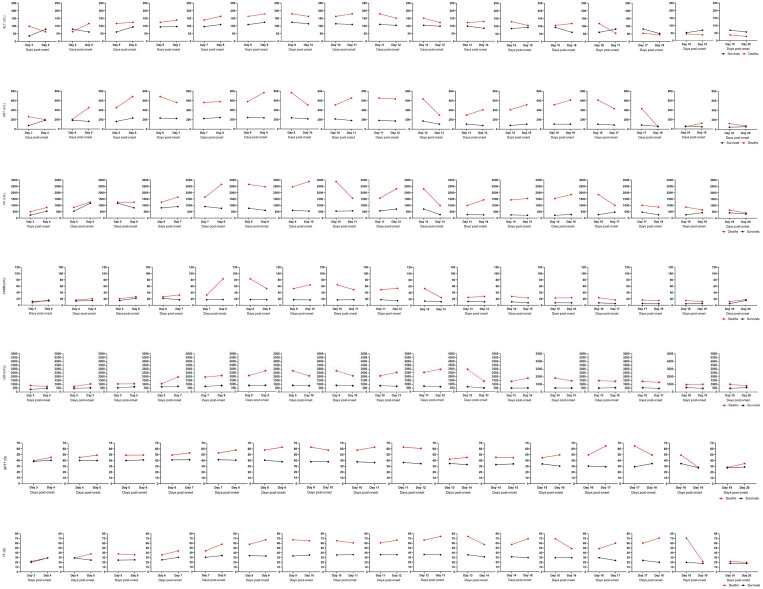
The change rate of risk factors in the course of the disease.

A dynamic statistical analysis of their laboratory parameters was conducted to monitor the progression of SFTS (Fig 3). In both the fatal and non-fatal groups, laboratory parameter values began to rise on Day 4, showed a marked increase by Day 7, and peaked around Day 10 before declining. The laboratory parameter values were consistently higher in the fatal group than in the non-fatal group. By Day 15, the parameter levels in both groups stabilized and converged to similar values. To further examine daily fluctuations, frequently occurring risk factors were identified, and slope plots were generated for their trends (Fig 4). These parameters showed an upward trajectory during the initial 10 days of illness, followed by a decline starting on Day 10 or Day 11. The steepest negative slope occurred on Days 12–13. After Day 15, the slope values were relatively low, indicating stabilization in laboratory parameter changes. The progression of SFTS over 20 days was accurately staged by analyzing the disease course across four aspects, resulting in the identification of five stages: initial stage (1–4 days), progressive stage (5–7 days), MOD stage (8–10 days), remission stage (11–14 days), and convalescence stage (15–20 days).

A line chart was made to compare the changes of the two groups of patients with the main risk factors in univariate analysis.

### Multi-organ dysfunction and SFTS management

#### Dynamic changes in multi-organ dysfunction.

Mortality risk factors were categorized into distinct biomarkers to monitor the evolution of MOD in patients with SFTS (Table 2 and Fig 3). These biomarkers reflect dysfunction across various systems:

- Myocardial injury markers: troponin, myoglobin, AST, CK, CKMB, LDH, HBDH, and BNP.
- Liver function parameters: ALT and AST.
- Infection indicators: PCT and CRP.
- Coagulation dysfunction markers: PT, APTT, and TT.
- Renal function markers: BUN and SCR
- Electrolyte imbalances, such as potassium, calcium, and chloride.

Patients with SFTS experience injuries across multiple organs, including the liver, kidneys, heart, coagulation system, and electrolyte balance. By correlating the timing of risk factors with organ dysfunction, the dynamic progression of MOD over the disease course was identified:

- **Initial stage (1–4 days)**: Myocardial, kidney, and liver injuries emerge, with myocardial injury persisting for an extended duration.
- **Progressive stage (5–7 days)**: Coagulation dysfunction and electrolyte imbalances become prominent.
- **Multi-organ dysfunction stage (8–10 days)**: This stage shows the most pronounced differences in patient outcomes between the fatal and non-fatal groups.
- **Remission stage (11–14 days)**: Although the patient’s condition begins to improve subsequently, the risk of death persists.
- **Convalescence stage (15–20 days)**: The patient’s condition stabilizes.

### Monitoring disease progression and dynamic treatment

The results highlight the dynamic progression of MOD in patients with SFTS. The impact of early IVIG treatment in patients with SFTS-associated myocarditis was analyzed to evaluate the clinical utility of accurate staging in monitoring and early intervention. The study involved 190 patients from Yijishan Hospital of Wannan Medical College, including 134 patients with myocarditis. Patients were divided into an IVIG-treated group and a non-IVIG group, with further stratification based on the timing of IVIG administration: 1–4 days, 5–7 days, and 8–10 days post-disease onset (Table 3). The findings revealed that IVIG treatment (p = 0.041) and the timing of its administration (p = 0.036) significantly influenced patient prognosis. These results underscore the importance of timely IVIG administration in improving outcomes for patients with SFTS-associated myocarditis.

**Table 3:** The effect of IVIG treatment on patients with SFTS.

Title	Group		χ^2^	P^a^
	In fatal group (%)	in non-fatal group (%)		
Whether to receive IVIG treatment				
IVIG group	12 (31.58)	49 (51.04)	4.158	0.041
Non-IVIG group	26 (68.42)	47 (48.96)		
Time of IVIG treatment, n, %				
Day 1 to Day 4	0 (0)	15 (30.61)	5.777	0.038
Day 5 to Day 7	10 (83.33)	30 (61.22)		
Day 8 to Day 10	2 (16.67)	4 (8.17)		

^a^χ^2^ tests were used to compare differences. p < 0.05 is considered statistically significant.

## Discussion

This multicenter study utilized dynamic analyses of clinical symptoms and laboratory parameters, combined with the progression of MOD, to establish a precise staging framework for SFTS. The disease course was categorized into five stages: initial stage (1–4 days), progressive stage (5–7 days), MOD stage (8–10 days), remission stage (11–14 days), and convalescence stage (15–20 days). The critical phase of SFTS typically spans approximately two weeks, with 85.71% of fatalities occurring within this period. Day 7 marks a pivotal point in the disease course, distinguishing the trajectories of the fatal and non-fatal groups. After this juncture, the extent and severity of MOD worsened in the fatal group, while the non-fatal group showed signs of stabilization. The progression stage presents a crucial window for disease control, as 46.75% of fatal outcomes occur during the MOD stage. By Day 11, clinical improvement becomes evident, underscoring the importance of timely intervention. The dynamic nature of organ damage in SFTS necessitates treatment strategies that evolve parallel to disease progression. Given the rapid disease trajectory, early evaluation and preemptive intervention are critical to mitigating progression. Delayed treatment risks rendering the condition unmanageable. Myocarditis is employed throughout the entire course of SFTS. Early administration of IVIG considerably reduces mortality in patients with associated myocarditis.

Among the 475 patients included in this study, 77 succumbed to this disease, yielding a mortality rate of 16.21%, consistent with prior research findings [1–5]. The disease course was categorized into a 20-day timeline based on time to death, dynamic risk factor analysis, laboratory parameter trends, and parameter slope changes. During the initial 1–4 days, most patients presented with fever, fatigue, headache, cough, and expectoration, with no fatalities reported. By Day 3, a limited number of risk factors emerged alongside minor laboratory abnormalities, signaling the onset of measurable disease progression. The progression phase of SFTS occurs within a 5–7 days period, marking the onset of fatalities. During this period, the number of risk factors increased significantly. A comparison of fatal and non-fatal groups revealed that the fatal group exhibits a more rapid rate of change in laboratory parameters, more significant variability, and accelerated disease progression. Day 7 was critical in the disease course, as patients’ conditions deteriorated markedly beyond this point. Severe complications, including impaired consciousness, DIC, MOD, and mortality, were more prevalent after this stage. The number of risk factors and abnormal laboratory parameter values peaked during this time, with the most pronounced changes observed between Days 7–9. Day 11 was a turning point, initiating the remission phase (Days 11–14). During this period, the extent and severity of MOD began to decline, and patients showed gradual clinical improvement. However, complete recovery was not yet achieved, as laboratory parameters remained abnormal; MOD and risk factors persisted. Clinical symptoms and signs resolved by 15–20 days, and laboratory parameters progressively returned to normal levels, indicating recovery. Prior studies by Gai et al. and Weng et al. [9,12] identified 1–7 days as the initial phase of SFTS and 7–13 days as the critical stage for disease progression. Our study focused on the dynamic changes occurring during the initial seven days. Many patients were in the early stage of the disease course (1–4 days) in the sample. Observing changes during this stage allowed us to monitor patient conditions before the disease progressed to more severe phases. Li et al. reported that surviving patients in the recovery phase (surviving patients at the recovery phase of disease group) demonstrated significantly lower viral loads and intermediate monocyte counts compared tothose in the acute phase (surviving patients at the acute phase of disease group)., corroborating our findings from a complementary perspective. [22]. Another key strength of this study is its dynamic univariate analysis, which combined risk factors with MOD to elucidate the progression of organ damage. The study confirmed that myocardial, renal, and hepatic damage predominantly occurred in the early stages, while coagulation and electrolyte abnormalities were more evident in the advanced stages. The nature and extent of MOD varied across disease stages. Although most organ dysfunctions resolved following the critical phase, myocardial dysfunction persisted the longest, spanning the entire course of SFTS in some cases.

Clinicians are increasingly concerned about how treatment strategies influence disease outcomes. Given the dynamic progression of SFTS and the evolving nature of multi-organ injury, treatment protocols must be adaptively adjusted based on the disease stage. The clinical presentation, along with the type and severity of MOD, varies significantly across different stages. During the MOD stage, the progression of SFTS is rapid, making intervention difficult by this point. Therefore, early and progressive stages warrant close monitoring and proactive management to prevent fatal outcomes. This study demonstrated that myocardial damage is present throughout the course of SFTS. Previous findings from our research indicated that myocarditis is closely associated with prognosis and represents a critical risk factor for mortality. A total of 134 patients with SFTS complicated by myocarditis were analyzed, focusing on the therapeutic role of IVIG and correlating treatment timing with disease stages. The results revealed that patients receiving IVIG in the early stage exhibited considerably higher survival rates, underscoring the efficacy of early intervention and reinforcing the clinical utility of the proposed staging framework.

In conclusion, our findings give clinicians a deeper understanding of SFTS progression and its dynamic changes, equipping them with critical markers for patient monitoring, prognosis determination, and timely intervention. Adjusting treatment strategies based on disease stage, particularly before the onset of MOD, may improve significant outcomes. However, this study’s scope is limited to clinical and laboratory parameters; it did not incorporate an evaluation of patient viral load, representing an area for future research.

## Methods

### Ethics approval and consent to participate

The study was conducted according to the guidelines of the Declaration of Helsinki. A statement to confirm that all experimental protocols were approved by the Ethics committee of Scientific Research and New Technology of Wannan Medical College Yijishan Hospital IRB (Research Proposal Notification IRB Review Decision [2021] [No. 24]). The requirement for informed consent was waived by the Ethics committee of Scientific Research and New Technology of Wannan Medical College Yijishan Hospital IRB because of the retrospective nature of the study.

### Patients

A retrospective analysis was performed on 475 patients diagnosed with SFTS admitted to the First Affiliated Hospital of Wannan Medical College and the First Affiliated Hospital of Nanjing Medical University between December 2020 and December 2023. The diagnosis of SFTS was confirmed by detecting SFTSV RNA using a real‐time reverse transcription‐polymerase chain reaction (RT-PCR) test. Inclusion criteria focused on adult patients with complete case data and hospital admission within two weeks of symptom onset. Exclusion criteria encompassed patients with blood disorders, cirrhosis, nephrotic syndrome, autoimmune diseases, chronic obstructive pulmonary disease, congenital heart defects, immunodeficiencies, or any history of malignancy. Patients with delayed hospitalization (over two weeks from onset) were excluded to ensure accurate disease course analysis.

### Data collection

Comprehensive data were gathered, including demographic details (age, sex, time from onset to admission, history of tick bites), clinical symptoms and signs, and laboratory parameters recorded within the first 20 days of disease onset. Laboratory measurements including white blood cell count, neutrophil count, lymphocyte count, monocyte count, red blood cell (RBC) count, hemoglobin, hematocrit (HCT), mean corpuscular volume (MCV), mean corpuscular hemoglobin (MCH), mean corpuscular hemoglobin concentration (MCHC), red cell distribution width (RDW), platelet count (PLT), mean platelet volume (MPV), platelet distribution width (PDW), reticulocyte count (RET), C-reactive protein (CRP), total protein, albumin, alanine aminotransferase (ALT), aspartate aminotransferase (AST), blood urea nitrogen (BUN), serum creatinine (SCR), cystatin-C, creatine kinase (CK), CK-MB, lactate dehydrogenase (LDH), hydroxybutyrate dehydrogenase (HBDH), potassium (K), sodium (Na), chloride (Cl), calcium (Ca), carbon dioxide combining power (CO_2_CP), amylase, lipase, prothrombin time (PT), activated partial thromboplastin time (APTT), thrombin time (TT), D-dimer (D-D), B-type natriuretic peptide (BNP), procalcitonin, troponin, myoglobin, ferritin, and interleukin-6.

### Statistical analysis

Statistical analysis was performed using GraphPad 9.0 (GraphPad Software) and Statistical Package for the Social Sciences (SPSS) statistical software (version 25.0, International Business Machines Corporation, New York, USA). Continuous variables are presented as means and standard deviations, while categorical variables are presented as frequencies and percentages. Group comparisons were made using the Mann–Whitney U test or chi-square (χ^2^) test, as appropriate. Survival analysis was conducted using Cox regression analysis. Statistical significance was set at a p-value of <0.05.

## Supporting information

S1 File Supporting data. (XLS)
